# Vitamin D Incorporation in Foods: Formulation Strategies, Stability, and Bioaccessibility as Affected by the Food Matrix

**DOI:** 10.3390/foods10091989

**Published:** 2021-08-25

**Authors:** Vera Lavelli, Paolo D’Incecco, Luisa Pellegrino

**Affiliations:** Department of Food, Environmental and Nutritional Sciences (DeFENS), University of Milan, 20133 Milan, Italy; paolo.dincecco@unimi.it (P.D.); luisa.pellegrino@unimi.it (L.P.)

**Keywords:** vitamin D, nanoemulsions, liposomes, casein micelles, protein nanocapsules, fortification, stability, bioaccessibility

## Abstract

Inadequate intake of vitamin D is a global health issue related to severe diseases, mainly involving subjects with dark skin pigmentation, patients affected by malnutrition, malabsorption syndromes, or obesity, and elderly people. Some foods fortified with vitamin D have been tested in vivo, but fortification strategies with a global outreach are still lacking. This review is focused on food fortification with vitamin D, with the aim to collect information on (a) formulation strategies; (b) stability during processing and storage; and (c) in vitro bioaccessibility. Approaches to add vitamin D to various foods were analyzed, including the use of free vitamin D, vitamin D loaded in simple and double nanoemulsions, liposomes, casein micelles, and protein nanocapsules. Numerous studies were reviewed to elucidate the impact of food technologies on vitamin D’s stability, and mechanisms that lead to degradation were identified—namely, acid-catalyzed isomerization, radical-induced oxidation, and photo-oxidation. There is, however, a lack of kinetic data that allow for the prediction of vitamin D’s stability under industrial processing conditions. The roles that lipids, proteins, fibers, and antioxidants play in vitamin bioaccessibility have been clarified in various studies, while future needs include the design of specific food matrices that simultaneously achieve a balance between the long-term stability, bioaccessibility and, ultimately, in vivo functionality of vitamin D.

## 1. Introduction

Vitamin D performs essential functions for human health, and concerns about its inadequate dietary intake have been expressed worldwide [1,2]. Vitamin D has two prevalent forms, i.e., cholecalciferol (vitamin D_3_) and ergocalciferol (vitamin D_2_). These vitamers are formed through the UV conversion of precursor compounds—namely, 7-dehydrocholesterol present in vertebrates for vitamin D_3_, and ergosterol present in fungi and some invertebrates for vitamin D_2_. The equivalence of the in vivo effectiveness of vitamin D_2_ and vitamin D_3_ has not been fully elucidated. In fact, some studies showed the same biological activity for both vitamers [3,4], while other studies suggested a higher bioavailability for vitamin D_3_ than for vitamin D_2_ [5,6,7]. Upon ingestion, both vitamin D_2_ and vitamin D_3_ are converted into the same biologically active form, i.e., 1,25-dihydroxyvitamin D (1,25(OH)2D). This latter compound binds to a nuclear receptor and modulates gene expression. Interestingly, the vitamin D receptor was found to be present in most cells in the body, and this finding supports the involvement of vitamin D in the prevention of multiple diseases [8,9]. In fact, vitamin D performs a fundamental role in the maintenance of skeletal calcium and phosphate balance, thus preventing rickets in children and osteomalacia, osteoporosis, and bone fractures in adults. Moreover, it has been associated with protection from the risk of common cancers, autoimmune diseases, hypertension, and infectious diseases [2]. The recommended dietary intake of vitamin D is 10, 15, and 20 µg/d for infants (<1 year), subjects aged 1–70 years, and subjects aged ˃ 70 years, respectively [10]. Observational studies led to the conclusion that vitamin D supplementation should be 2–3 times higher for obese subjects and 1.5 times higher for overweight subjects, relative to normal-weight subjects [11]. Moreover, for people at risk of vitamin D deficiency, the recommended daily dose of vitamin D is 50 µg/d or higher, depending on age [10]. The recognized biomarker for vitamin D status is serum 25-hydroxyvitamin D (25(OH)D) level. Some panels of experts indicate that vitamin D deficiency corresponds to a (25(OH)D) level below 50 nmol/L, vitamin D insufficiency to a level between 50 and 75 nmol/L, and vitamin D toxicity to levels above 500 nmol/L [10]. However, other panels of experts indicated that the cutoff below which the risk of clinical vitamin D deficiency increases corresponds to a serum level of 25(OH)D < 30 nmol/L [12]. Populations at risk of vitamin D deficiency include subjects with dark skin pigmentation, subjects affected by malnutrition, malabsorption syndromes, or obesity, and elderly people [9]. Vitamin D insufficiency is also common. In fact, a survey throughout Europe has shown that the intake of vitamin D is inadequate for 77–100% of adults (19–64 years old) and for 55–100% of elderly adults (>64 years old) [13]. Compared to the USA, Vitamin D intake from food in Europe is lower [14], which can be attributed to the higher availability of foods fortified with vitamin D in the USA than in Europe [1]. Indeed, food fortification with vitamin D is considered a fundamental strategy to fight vitamin D malnutrition and its associated diseases [1,3,15].

Foods fortified with vitamin D—especially bread and dairy products—have been assessed in vivo for their efficacy in raising the level of 25(OH)D and improving health in different target populations (Table 1). In both low-fiber white bread and high-fiber sourdough rye bread, fortification with a water-dispersible form of vitamin D_3_ providing approximately 10 µg/d was as effective as vitamin D_3_ supplements in increasing serum 25(OH)D concentration in healthy women aged 25–45 years [16]. The production of bread with UV-irradiated yeast as a source of 25 µg/d of vitamin D_2_ did not affect serum 25(OH)D concentration in healthy women aged 20–37 years, while supplements of both vitamin D_2_ and vitamin D_3_ resulted in a positive effect [17]. Conversely, vitamin D_2_ from UV-irradiated mushroom formulated in a soup was as effective as vitamin D_2_ supplements in increasing serum 25(OH)D concentration in healthy subjects younger than 45 years, but the amount provided was high, equal to 700 µg/d [18]. In Ca^++^-enriched orange juice, water-dispersible forms of either vitamin D_2_ or vitamin D_3_ in a dose of 25 µg/d were equally bioavailable as the respective supplements in adult subjects aged 18–84 years [4]. The impact of Ca^++^ enrichment along with vitamin D_2_ was not investigated, because a control without Ca^++^ addition was not included [4].

Reduced-fat milk enriched with Ca^++^ was an effective matrix for vitamin D_3_ fortification, when administered at a level of 20 µg/d to healthy male subjects aged 50–87 years, causing increased serum 25(OH)D concentration and reduced bone loss, but no information was provided on the incorporated form of vitamin D_3_ [19]. In a subsequent study, fortification of Ca^++^-enriched milk with a non-specified form of vitamin D_3_, as well as fortification of Ca^++^-enriched orange juice, were designed for healthy children aged 9–12 years, and were found to be effective in increasing serum 25(OH) concentration, while serum osteocalcin and intact parathyroid hormone were not affected [20]. However, in these latter two studies, foods enriched in Ca^++^ and vitamin D were also considered, but the impact of Ca^++^ enrichment was not investigated [19,20].

Contradictory results were obtained by applying a water-dispersible form of vitamin D_3_ in cheese, but the supplied amounts were different [21,22]. In fact, in one study involving healthy, ≥60-year-old subjects, no effects on 25(OH)D, parathyroid hormone, or osteocalcin serum concentrations were observed upon the consumption of vitamin-D_3_-fortified cheese supplying 15 µg/d [21], while in another study involving healthy subjects aged 18–60 years, an increase in serum 25(OH)D concentration was observed along with a decreased serum parathyroid hormone upon consumption of either regular Cheddar cheese (obtained from milk having 3.8% fat content) or reduced-fat cheese (obtained from skim milk having 0.8% fat content), both supplying 100 µg/d of vitamin D_3_ [22].

**Table 1:** In vivo studies on the effects of vitamin-D-fortified foods.

Fortified Food	Subjects and Duration	Dose (µg/d)	Outcome	Ref.
Water-dispersible vitamin D_3_ in low-fiber wheat bread	25–45 years, healthy women *n* = 41, 1 month	10.8	Both vitamin-D_3_-fortified breads increased serum 25(OH)D as effectively as vitamin D_3_ supplement.	[16]
Water-dispersible vitamin D_3_ in high-fiber sourdough rye bread		12.3		
Vitamin D_2_ from UV-irradiated yeast in bread	20–37 years, healthy women *n* = 33, 2 months	25	Vitamin D_2_ from UV-irradiated yeast in bread did not raise serum 25(OH)D.	[17]
Vitamin D_2_ from UV-irradiated mushrooms in soup	<45 years, healthy *n* = 26, 1 month	700	Vitamin-D_2_-fortified soup increased serum 25(OH)D as effectively as vitamin D_2_ supplement.	[18]
Water-dispersible vitamin D_3_ in Ca^++^-enriched orange juice (Ca^++^ dose: 350 mg/d)	18–84 years, healthy *n* = 105, 3 months	25	Vitamin D_2_ and vitamin D_3_ were equally bioavailable in Ca^++^-enriched orange juice and capsules.	[4]
Water-dispersible vitamin D_2_ in Ca^++^-enriched orange juice (Ca^++^ dose: 350 mg/d)				
Vitamin D_3_ non-specified in Ca^++^-enriched reduced-fat milk (Ca^++^ dose: 1000 mg/d)	50–87 years, healthy men *n* = 149, 2 years	20	Vitamin-D_3_- and Ca^++^-fortified milk increased serum 25(OH)D and reduced bone loss.	[19]
Vitamin D_3_ non-specified in Ca^++^-enriched orange juice (Ca^++^ dose: 500 mg/d)	9–12 years, healthy *n* = 410, 3 months	2.5	Vitamin-D_3_-fortified milk and orange juice increased serum 25(OH). Serum OC and PHT were not affected.	[20]
Vitamin D_3_ non-specified in Ca^++^-enriched milk (Ca^++^ dose: 500 mg/d)				
Water-dispersible vitamin D_3_ in cheese	≥60 years, healthy *n* = 110, 2 months	15	Vitamin-D_3_-fortified cheese had no effect on serum 25(OH)D, OC and PHT.	[21]
Water-dispersible vitamin D_3_ in Cheddar cheese	18–60 years, healthy *n* = 30, 2 months	100 *	Both vitamin-D_3_-fortified cheeses increased serum 25(OH)D and decreased serum PHT as vitamin D_3_ supplement	[22]
Vitamin D_3_ non-specified in regular yogurt (Ca^++^ dose: 150 mg/d)	30–60 years, diabetic *n* = 90, 3 months	25	Both vitamin-D_3_-fortified regular yogurt and Ca^++^-enriched yogurt increased serum 25(OH)D and improved glycemic status.	[23]
Vitamin D_3_ non-specified in Ca^++^ enriched yogurt (Ca^++^ dose: 250 mg/d)				
Vitamin D_3_ non-specified in Ca^++^ enriched yogurt (Ca^++^ dose: 800 mg/d)	≥65 years, healthy women *n* = 20, 3 months	10	Vitamin-D_3_-fortified yogurt increased serum 25(OH)D, and maintained cognitive performance.	[24]
Water-dispersible vitamin D_3_ in yogurt	37–47 years, pre-diabetic *n* = 60, 3 months	25	Vitamin-D_3_-fortified yogurt increased serum 25(OH)D as effectively as vitamin D_3_ supplement, and improved serum lipid profile.	[25]
Vitamin D_3_ in casein micelles in low-fat yogurt	18–61 years, healthy *n* = 87, single intake	1250	Both vitamin D_3_ carriers in yogurt increased serum 25(OH)D.	[26]
Vitamin D_3_ in emulsion in low-fat yogurt				

*: Equivalent to a daily dose (fortified food was ingested in one weekly dose); PHT: parathyroid hormone; OC: osteocalcin.

Various approaches have been applied for yogurt fortification with vitamin D_3_. In one study, a non-specified form of vitamin D_3_ was added alone or in combination with Ca^++^ in yogurt, and both formulations supplying 25 µg/d were effective in raising serum 25(OH)D levels and improving the glycemic status of diabetic subjects aged 30–60 years. Hence, the combined effect of Ca^++^ and vitamin D enrichment of the food matrix seemed not to be relevant [23]. Positive effects on serum 25(OH)D level and in the maintenance of cognitive performance were also observed upon consumption of yogurt enriched with Ca^++^ and 10 µg/d of a non-specified form of vitamin D_3_ by a population of healthy elderly women (≥65 years), while vitamin D enrichment of yogurt with regular Ca^++^ content was not considered [24]. In another approach, the water-dispersible form of vitamin D_3_ was used to fortify yogurt, and proved to be effective in increasing serum 25(OH)D levels and improving the serum lipid profiles of pre-diabetic subjects aged 37–47 years [25], when administered at a dose of 25 µg/d. Casein micelles or polysorbate/Tween-80 emulsions were designed as carriers to formulate vitamin D_3_ in low-fat yogurt, and both formulations were found to be effective in raising serum 25(OH)D in healthy subjects aged 18–61 years, but the dose administered was equal to 1250 µg/d [26]—well above the recommended daily dose.

The above-reported in vivo studies cannot be compared because of the different target populations involved, daily doses administered, and durations of the interventions. However, in general they support the efficacy of fortification of various food matrices as a strategy to manage inadequate vitamin D intake. Nevertheless, in order to apply a vitamin D fortification strategy, further issues need to be considered. Firstly, the form in which vitamin D was administered is generally unknown or lacks relevant information, and the dosage applied was higher than the recommended daily dose. Hence, no information on the cost-effectiveness of the fortification strategy can be derived. Moreover, in most in vivo studies, food fortification was performed by adding vitamin D to the processed product just before the administration, while no information is provided on vitamin D stability when addition is carried out prior to food processing, packaging, and long-term storage. In vitro modelling studies can provide in-depth information on vitamin D’s stability and bioaccessibility. Hence, this review article is focused on food fortification with vitamin D, with the aim to collect information on (a) the formulation strategies of vitamin D in foods; (b) the stability of vitamin D in fortified foods during processing and storage; and (c) the in vitro bioaccessibility of vitamin D from the fortified foods. The ultimate aim of this study is to support the design of an efficient technology for the vitamin D fortification of foods.

## 2. Vitamin D Fortified Foods: Formulation Strategies

Vitamin D_3_ obtained at the industrial level is generally used for food fortification. The free form of vitamin D_3_ has likely been incorporated in the food matrices evaluated in some in vivo studies, although this is not specifically indicated [19,20,23,24]. Free vitamin D_3_ has also been used in various in vitro studies [27,28,29,30,31,32,33], while free vitamin D_2_ has been considered in only a few in vitro studies [31].

Irradiated yeasts and mushrooms have been used directly as a source of vitamin D_2_ in fortified foods for in vivo [17,18] and in vitro studies [30]. The application of the unprotected compound in food formulation has the major drawbacks of non-homogeneous dispersion in the matrix, uncontrolled stability, and fast release. Moreover, the application of vitamin D_2_ entrapped in yeast cells was not effective in vivo [17], while that entrapped in mushrooms was effective in vivo only at very high dosages [18].

Vitamin D is a large sterol molecule. Considering its low water solubility and susceptibility to oxidative or heat degradation, suitable delivery systems have been studied for food fortification (Table 2). In brief, water-dispersible vitamin premixes—often containing an emulsifier—and oil-based systems have been proposed, depending on the characteristics of the target food. A commercial water-soluble form of vitamin D containing propylene glycol and polysorbate 80 (Vitex-D, Kingsway Chocolate Co. Ltd., Bunge Foods, Mississauga, ON, Canada) was used in one in vivo [22] and in some in vitro studies [34,35], but no information was provided on the formulation characteristics, such as its particle size distribution, which affects its bioaccessibility [36]. Other in vivo [4,16,20,25] or in vitro [37,38,39] studies reported the use of water-dispersible forms of vitamin D_3_ or vitamin D_2_, but no details were provided on composition or particle size distribution.

A strategy to develop value-added fortified foods is the design of specific nano- or micro-structures (Table 2) that enable the homogeneous distribution of bioactive compounds in the food matrix and improve stability and bioavailability [40]. It is worth noting that nano- and micro-structures can potentially be affected by pH, ionic strength, and heat treatments; hence, it is fundamental to test their stability during food processing [41].

It must be underlined that many of the carrier systems developed for vitamin D delivery look promising, but have not found application at the industrial level so far. This review only focuses on studies including a food product application—namely, lipid-based encapsulation systems [42,43,44,45,46,47,48,49,50], which were considered in vitro studies, and protein complexes, which were considered in studies performed both in vivo [26] and in vitro [51,52].

Lipid-based encapsulation systems used for food fortification with vitamin D include oil-in-water (O/W) nanoemulsions [36,43,45,46,47]; double water-in-oil-in-water (W_1_/O/W_2_) emulsions [48], and liposomes [50]. All of these structures can be affected by chemical instability due to lipid oxidation and physical instability due to their tendency to phase separation.

O/W emulsions are structures with a lipophilic core where a lipophilic bioactive compound is entrapped, a hydrophilic shell (aqueous phase), and an amphiphilic interface consisting of the emulsifier/surfactant [53]. Emulsion particles move randomly in the presence of gravitational and Brownian motion forces acting on the droplets in the medium. The main instability processes are flocculation, coalescence, and creaming (or sedimentation), which ultimately lead to complete separation of the two constituting phases. In nanoemulsions, small particle size (d < 200 nm) creates low susceptibility to gravitational separation due to the dominant Brownian forces [53]. The zeta potential (ζ-potential) of dispersed oil droplets is also related to emulsion stability, because levels close to zero promote aggregation due to the absence of charge repulsions [54]. In one approach, vitamin D_2_ emulsions were prepared using corn oil, Tween^®^ 80, and phosphate buffer; the amount of vitamin D_2_ in the oil phase was 50 mg/100 g of oil. By blending these components at a high speed (10,000 rpm), a large emulsion was obtained with a volume-weighted mean diameter of 14.5 μm and ζ-potential of −13.8 mV; by passing the large emulsion through a Microfluidizer operating at 6000 psi, a medium emulsion was obtained with a volume-weighted mean diameter of 0.53 μm, and further processing at 15,000 psi resulted in a small emulsion with a volume-weighted mean diameter of 0.11 μm, with little variation in the ζ-potential [36]. Hence, it was possible to modulate emulsions’ nanostructure by varying the energy input of the process. The droplets should have had a low net charge because they were stabilized by a non-ionic surfactant; however, a negative surface charge was observed and attributed to the presence of anionic impurities such as free fatty acids in either the surfactant or the oil phase [36]. The negative charge is expected to inhibit particle aggregation, but the emulsions were not tested in food matrices [36]. In other studies, emulsions were prepared using vitamin D_3_, flaxseed oil, calcium caseinate, and distilled water, +/− soy lecithin, but the amount of vitamin D_3_ was not specified. No information was provided on the particle size distribution and ζ-potential; however, physical stability was proven up to 6 d of storage in the absence of soy lecithin and 15 d of storage in the presence of soy lecithin; afterwards, the appearance of serum indicated that casein–casein interactions occurred. The adsorption of lecithin to the oil–water interface reduced the interactions between proteins and led to a slow gelation of the emulsion, which was then added to cheese [43]. In another approach, a nanoemulsion of vitamin D_3_ was created by combining vitamin D_3_ in corn oil and the surfactant (quillaja saponin) into phosphate buffer solution (5 mM, pH 7); the amount of vitamin D_3_ was 4 g/100 g of the oil phase. The mean particle diameter of the vitamin-enriched nanoemulsions was 0.14 μm, the pH was 4.74, and the surface charge was negative (–74.1 mV). Due to the strong repulsions between the negative charges on the surface, the nanoemulsion was stable for 7 d of storage, and was found to be suitable for the fortification of almond milk [47].

The W_1_/O/W_2_ double emulsions have a water-continuous system containing oil droplets with smaller water droplets dispersed within. Double emulsions can simultaneously encapsulate both hydrophobic and hydrophilic compounds. Moreover, double emulsions can have a reduced fat content compared to O/W emulsions. As with the single emulsions, double emulsions can undergo flocculation, coalescence, and creaming (or sedimentation); additional instability processes include swelling and rupture of the inner droplets induced by osmotic transport between the two aqueous phases. Hence, there should be a balance between the osmotic pressure of the inner and outer aqueous regions [54]. In one study, W_1_/O/W_2_ double emulsions containing vitamin D_3_ were considered; the W_1_ phase was prepared by adding black chokeberry phenolic extract, folic acid, vitamin B_12_, and vitamin C; the O phase consisted of polyglycerol polyricinoleate dispersed in rapeseed oil containing vitamin D_3_ and vitamin A; and the W_2_ phase was a milk protein solution. The emulsification process included the preparation of the primary emulsion at a high speed (15,000 rpm) and the preparation of the secondary emulsion at a low speed (11,000 rpm). The amount of vitamin D_3_ was 7 mg/100 g of oil, and encapsulation efficiency was 98.52%. The resulting W_1_/O/W_2_ emulsion had a monomodal particle size distribution with a volume moment-weighted mean diameter of 37.65 µm, and was physically stable for 30 d [48]. This W_1_/O/W_2_ double emulsion was then added to yogurt [49].

Liposomes are vesicles with an aqueous core surrounded by one or more amphiphilic molecules—such as polar lipids—that produce a bilayer structure. Hydrophobic substances can be entrapped within the hydrophobic bilayer [55]. Phospholipids are often used as amphiphilic molecules for liposome preparation. The small radius of the curvature of the vesicles generates instability because it disrupts the regular packing of the phospholipid bilayer and imposes constraints on the phospholipid molecules [56]. One of the key parameters for the liposome structure is the transition temperature, where the liposomes lose their ordered packing structure due to melting of the hydrocarbon chains, i.e., they undergo a phase transition from gel to liquid-crystalline. Since natural phospholipids contain hydrocarbon chains that differ in length, phase transition occurs over a wide temperature range. Physical instability of liposomes may arise from merging, which results in increased particle size. Cholesterol is generally added to the phospholipid structure at a volume of over 20% because it decreases the transition temperature and increases stability [55]. Lipophilic bioactive compounds that are positioned in the lipid bilayer of liposomes, such as vitamin D, can alter the interactions between hydrocarbon chains and, hence, affect bilayer stability. In one study, liposomes loaded with vitamin D_3_ were added to milk intended for producing fortified cheese, but a commercial liposome premix was used and no information was provided on liposome structure [57]. In another study, liposomes loaded with vitamin D_3_ were applied in chocolate. Phospholipids, cholesterol, and vitamin D_3_ were used in a 3:1:1 ratio; hence, vitamin D_3_ constituted 20 g/100 g of the lipid phase. Liposomes were obtained by film dispersion–homogenization. Loading efficiency for vitamin D_3_ was 68.2%. The resulting liposomes had a monomodal distribution, with an equivalent volume diameter at 50% cumulative volume of 354.91 nm and a ζ-potential of −27.62 mV; however, no information on stability was provided [50].

Hydrophobic molecules can also bind to proteins via hydrophobic interactions. A consensus exists on the role of some well-structured proteins, such as milk and soy proteins, as natural nanocarriers of hydrophobic bioactive compounds [58,59,60,61,62,63]. However, the loading capacity of these structures is likely low, being targeted at the naturally occurring levels of bioactive compounds. Thus, protein-based nanocarriers have been developed as vehicles of vitamin D at the target levels of fortification in food, with casein being the most frequently involved protein.

Complexation of vitamin D with whey proteins proved to increase the stability of the vitamin under light and prolonged storage, compared to the free form, but no data obtained on real foods were given [59,60]. Compared to native casein micelles, sodium or calcium caseinates are more water soluble, and single casein molecules still maintain the capacity for self-assembly due to their amphiphilic nature. On this basis, the so-called reassembled casein micelles (rCMs) have been studied and shown to represent microencapsulation structures that potentially guarantee enhanced stability of vitamin D during subsequent food processing. The vitamin-loading capacity of rCMs essentially depends on the content of calcium, phosphate, and citrate added in the formulation. By optimizing these parameters, a loading of 1.38–1.46 mg/100 mg casein was achieved for vitamin D, with particles of rCMs showing a monomodal distribution and average volume-weighted particle sizes of 16–19 µm, depending on phosphate concentration [61]. However, aqueous suspensions of the rCMs showed D(0.5) (diameter where 50% of the particle size distribution is above and 50% is below) values around 12–13 µm, i.e., much larger than those of native casein micelles (50–700 nm), indicating that rCMs may merge into large particles due to the high phosphate content (4.9 mM). Other authors [26], who prepared vitamin-D-loaded rCMs with the same approach, achieving a loading of 1.6 mg vitamin D_3_/100 mg casein, adopted a homogenization step of the mixture, and obtained a bimodal volume-weighted particle size distribution, with a large population that had an average volume moment-weighted diameter of 89 nm, and a smaller population around 277 nm in diameter. The efficacy of casein complexes and rCMs in protecting the associated vitamin D_2_ from degradation was compared in fluid milk processed at the laboratory scale [51]. Disruption and subsequent reassembly of casein micelles with increased affinity for vitamin D was also attained using high-pressure (> 400 MPa) treatments at selected temperatures [62]. Large rCMs (278 nm average hydrodynamic diameter) with a vitamin D_2_ loading of 10.4 µg/mg casein were obtained by treating a dispersion of native micelles at 600 MPa at 50 °C. These structures were claimed to be suitable for the enrichment of low-fat dairy products, since they maintain the original properties [62].

**Table 2:** Formulation of vitamin D for food fortification.

Structures and Vitamin D Loading	Components	PSD and Average Size	ζ-Potential	Ref.
O/W emulsions S, small; M, medium; L, large 50 mg vitamin D_2_/100 g oil	Vitamin D_2_, corn oil, Tween^®^ 80, phosphate buffer	Monomodal S: 0.11 μm, M: 0.53 μm, L: 14.5 μm (volume moment-weighted mean diameter)	S: −12.4 mV, M: −14.1 mV, L: −13.8 mV	[36]
O/W nanoemulsion 4 g vitamin D_3_ /100 g oil	Vitamin D_3_, corn oil, quillaja saponin, phosphate buffer	Monomodal 0.14 µm (mean particle diameter)	−36.4 mV	[47]
W_1_/O/W_2_ double emulsion 7 mg vitamin D_3_/100 g oil	Vitamin D_3_, vitamin A, vitamin B_12_, vitamin C, chokeberry extract, folic acid, rapeseed oil, polyglycerol polyricinoleate, milk protein, sodium chloride, water	Monomodal 37.65 µm (volume moment-weighted mean diameter)		[48]
Liposomes 20 g vitamin D_3_/100 g lipid phase	Vitamin D_3_, phospholipids, cholesterol in ethanol, distilled water	Monomodal 354.91 nm (equivalent volume diameter at 50% cumulative volume)	−27.62 mV	[50]
rCMs: reassembled casein micelles 1.38–1.46 mg vitamin D_3_/100 mg casein	Vitamin D_3_ in ethanol, sodium caseinate in water, dipotassium hydrogen phosphate, potassium citrate, calcium chloride	Monomodal from 16.10 to 19.04 µm (average volume-weighted particle sizes)	from −17.3 to −17.8 mV	[61]
rCMs: reassembled casein micelles 1.6 mg vitamin D_3_/100 mg casein	Vitamin D_3_ in ethanol, sodium caseinate in water, dipotassium hydrogen phosphate, tripotassium citrate, calcium chloride	Bimodal 89 nm and 277 nm (volume moment-weighted mean diameter)		[26]
rCMs: reassembled casein micelles 1 µg vitamin D_2_/100 mg casein	Vitamin D_2_ in ethanol, micellar casein in sweet whey permeate	Monomodal from 145 to 303 nm (mean hydrodynamic diameter)		[62]
β-Conglycinin nanoparticles 10 µg vitamin D_3_/100 mg β-conglycinin	Vitamin D_3_ in ethanol, β-conglycinin, phosphate buffer	Bimodal 31 nm and 120 nm (volume weighted average particle diameter)		[63]
Zein nanocapsules vitamin D_3_ loading not specified	Vitamin D_3_ in ethanol, zein, Tween^®^ 80	Monomodal 185.7 ± 2.10 nm (Z-average size)	24.5 mV	[52]

PSD: particle size distribution.

Due to their wider consumer acceptance and lower costs compared to animal proteins, plant proteins were also studied as vehicles for numerous bioactive compounds. Vitamin D_3_ was encapsulated in soybean β-conglycinin nanoparticles, leading to a bimodal distribution: a large population (90% of the particle volume) with an average volume moment-weighted diameter of 31 nm, and the remaining 10% with a diameter around 120 nm [63]. These nanoparticles were claimed to be suitable for the fortification of both foods and clear beverages [63]. Zein—a major protein from corn—was successfully used to produce nanoparticles that were loaded with vitamin D_3_, attaining a 97.27% encapsulation efficiency [52]. In this case, nanoparticles had a Z-average size of 185.7 nm and, upon observation via transmission electron microscopy (TEM), showed a smooth surface. The ζ-potential of 24.5 mV was attributed to the cationic character of zein, because the value was similar (21.2 mV) in the unloaded zein nanoparticles. A jelly based on the pulp of *Acca sellowiana*, xylitol, and pectin was then fortified with the nanoparticles [52].

The formulations described in Table 2 were generally tested in model foods: the O/W emulsions were added to cheese [43] and almond milk [47], the W_1_/O/W_2_ double emulsions were added to yogurt [49], liposomes were added to cheese [56] and chocolate [50], the rCMs were added to milk [51], and the zein nanocapsules were assessed in a model jelly food [52]. Although these model foods were prepared at the laboratory scale, they proved that the designed formulations could persist after processing and, thus, pave the way for applications in real food systems.

## 3. Vitamin D in Fortified Foods: Yield and Stability

The *cis*-triene configuration of vitamin D makes it potentially sensitive to isomerization and oxidation. Nevertheless, purified vitamin D_3_ was found to be stable at neutral pH, in air, at ambient temperature [64]. Accordingly, a differential calorimetric study performed on the purified vitamins D_2_ and D_3_ revealed that the onset temperatures for their thermal decomposition are about 166 and 156 °C, respectively, and their activation energy values are 141 and 131 kJ mol^−1^, respectively [65]. However, vitamin D’s stability can be affected by the food matrix components (Table 3).

In the presence of acids, vitamin D_3_ isomerizes, and its acid-catalyzed isomerization product—i.e., isotachysterol—is very oxygen-sensitive [63,66]. Moreover, vitamin D_3_ degradation is triggered by unsaturated lipid oxidation [29,67]. The reaction pattern for isomerization and oxidation of vitamin D_3_ does not involve the side chain that differs between vitamin D_2_ and D_3_; hence, these reactions most likely occur in vitamin D_2_ as well.

Considering that vitamin D is often formulated as an oil phase before being used in food processing, some studies have investigated vitamin D’s stability in bulk oils. Soybean oil packed in polyethylene terephthalate bottles was used as a model matrix to study the effects of oxidative status, antioxidant content (α-tocopherol), and exposure to light and oxygen on vitamin D_3_ degradation at 30 °C. This approach confirmed that the degradation of vitamin D_3_ occurs via oxidation, and the factors affecting degradation were, in decreasing order: storage time, exposure to light, and initial oxidative status of the oil, whereas α-tocopherol content played a protective role. As a result, vitamin D_3_ retention after storage at 30 °C for 50 d varied in the range 32–76% [29]. Sunflower oil was also used as a medium to study both vitamin D_3_ and vitamin D_2_ degradation at 100 and 210 °C, and both isomers were found to degrade at a similar rate upon heating [31].

Formulation of vitamin D_3_ in lipids with a low degree of unsaturation led to higher stability. In fact, both free vitamin D_3_ and liposomes made with vitamin D_3_, phospholipids, and cholesterol added to white chocolate after conching resulted in total recovery of the vitamin in chocolate upon 120 d of storage at 25 °C [50]. In contrast, a short-term stability of liposomes was observed in cheese upon ripening, leading to a progressive degradation of the loaded vitamin D [57].

Whole wheat flour was also considered for vitamin D_3_ incorporation, which is a strategy to develop various fortified bakery products [68]. In dry and intermediate-moisture foods, chemical stability is critically dependent on water activity (a_w_). For oxygen-sensitive compounds, stability decreases with increasing a_w_ above the monomolecular moisture content, due to a decrease in the viscosity of the food matrix and increase in the molecular mobility, accelerating oxidation [69]. Accordingly, the half-times for vitamin D_3_ added to whole wheat flour at 25 °C were found to be 173, 169, and 116 d at a_w_ levels of 0.33, 0.63, and 0.93, respectively. The stability of vitamin D_3_ greatly decreases with increasing temperature to 45 °C, with half-times for degradation of 87, 77, and 63 d at a_w_ of 0.33, 0.63, and 0.93, respectively [68].

Dry irradiated mushroom powder could represent a source of vitamin D_2_ for a variety of foods [70]; in this matrix, vitamin D_2_ stability was also found to decrease with increasing a_w_ from 0.11 to 0.75 [71]. The activation energy found for vitamin D_2_ degradation in mushroom powder at a_w_ 0.33 was 36.7 kJ/mol, which is close to the activation energy for oxygen diffusion in unsaturated lipids—i.e., 24 kJ/mol—and to the activation energies found for the free radical chain reactions in triglycerides—i.e., 34.0–37.3 kJ/mol [72]; hence, autoxidation was assumed to be the mechanism by which vitamin D_2_ was lost [71]. The half-life of vitamin D_2_ at a_w_ 0.33, calculated on the basis of the activation energy provided by this latter study, was 175 d at 25 °C, which is the same as that found for vitamin D_3_ in whole wheat flour under the same temperature and a_w_ conditions [68]. Conversely, in fresh irradiated mushrooms, vitamin D_2′_s half-life was approximately 7 d at both 4 °C and −14 °C [73]; hence, storage at −20 °C is necessary to preserve vitamin D_2_ in fresh mushroom [74].

**Table 3:** Recovery of vitamin D in fortified foods after processing and storage.

Fortified Food	Vitamin D Content (μg/100 g)	Main Processing Steps	Vitamin D Yield (%)	Ref.
Free vitamin D_3_ in soybean oil	700	Storage in PET bottles at 30 °C for 50 d in the dark	56–76 (S)	[29]
		Storage in PET bottles at 30 °C for 50 d under natural light	32–39 (S)	
Free vitamin D_3_ in sunflower oil	14.5	Heating at 110 °C for 30 min	85 (P)	[31]
		Heating at 210 °C for 10 min	79 (P)	
Free vitamin D_2_ in sunflower oil	11.2	Heating at 110 °C for 30 min	89 (P)	
		Heating at 210 °C for 10 min	76 (P)	
Vitamin D_3_ in liposomes in white chocolate	30	Three-step tempering process (33 °C–35 °C, 24 °C–25 °C, and 25 °C–26 °C), storage at 25 °C for 120 d	100 (S)	[50]
Free vitamin D_3_ in white chocolate			99 (S)	
Free vitamin D_3_ in whole wheat flour	nd	Storage at a_w_ 0.33 at 25 °C for 173 d	50 (S)	[68]
		Storage at a_w_ 0.93 at 25 °C for 116 d	50 (S)	
		Storage at a_w_ 0.33 at 45 °C for 87 d	50 (S)	
		Storage at a_w_ 0.93 at 45 °C for 63 d	50 (S)	
Vitamin D_2_ in dry mushroom powder	4420	Storage at a_w_ 0.33 at 25 °C for 175 d	50 (S)	[71]
		Storage at a_w_ 0.33 at 30 °C for 136 d	50 (S)	
		Storage at a_w_ 0.33 at 45 °C for 55 d	50 (S)	
Free vitamin D_3_ in wheat bread	10.8	Baking at 170 °C for 60 min	85 (P)	[28]
Free vitamin D_3_ in rye bread	8.7		69 (P)	
Vitamin D_2_ yeast in wheat bread	9.5		85 (P)	
Vitamin D_2_ yeast in rye bread	8.5		73 (P)	
Free vitamin D_3_ in wheat bread	40.2	Baking at 200 °C for 20 min	40.2 (P)	[68]
Free vitamin D_3_ in cookies	64	Baking at 200–205 °C for 12 min	65.3 (P)	
Free vitamin D_3_ in milk	12	Steam injection (95 °C), spray-drying at 149 °C, fluid-bed finish at 107 °C	100 (P)	[27]
Water-dispersible vitamin D_3_ in milk	100	Pasteurization at 73 °C for 15 s, homogenization at 13.8/3.4 MPa, storage at 4 °C for 21 d	100 (P, S)	[37]
Water-dispersible vitamin D_2_ in milk	2	Pasteurization at 63 °C for 30 min, storage in glass bottles at 4°C for 7 d in the dark	100 (P, S)	[39]
		Pasteurization at 63 °C for 30 min, storage in glass bottles at 4 °C for 32 h under light	100 (P, S)	
		Pasteurization at 63 °C for 30 min, storage in PET pouches at 4 °C for 7 d in the dark	~ 90 (P, S)	
		Pasteurization at 63 °C for 30 min, storage in PET pouches at 4 °C for 32 h under light	~ 90 (P, S)	
		Sterilization at 121 °C for 15 min	100 (P)	
Vitamin D_2_ in casein complexes in cow and buffalo milk (1:1)	1.25	Pasteurization at 63 °C for 30 min, storage in glass bottles at 4 °C for 7 d under light	~ 95 (P), ~ 70 (S)	[51]
		Pasteurization at 63 °C for 30 min, storage in LDPE pouches at 4 °C for 7 d under light	~ 95 (P), ~ 65 (S)	
		Sterilization at 121 °C for 15 min	~ 75 (P)	
Free vitamin D_2_ in cow and buffalo milk (1:1)	1.25	Pasteurization at 63 °C for 30 min, storage in glass bottles at 4 °C for 7 d under light	90 (P), 45 (S)	
		Pasteurization at 63 °C for 30 min, storage in LDPE pouches at 4 °C for 7 d under light	90 (P), 45 (S)	
		Sterilization at 121 °C for 15 min	67 (P)	
Water-dispersible vitamin D_3_ in Cheddar (milk fat 3.9%)	nd	Starter addition, rennet addition, salting, pressing, vacuum packaging, storage at 4° C for 84 d	90.4 (P) 100 (S)	[34]
Free vitamin D_3_ in Cheddar (milk fat 3.9%)			86.7 (P) 93.7% (S)	
Water-dispersible vitamin D_3_ in Cheddar (milk fat 3.8%)	2085	Milk pasteurization 72°C for 16 s, starter addition, rennet addition, salting, pressing, vacuum packaging, storage at 4 °C for 365 d	91 (P) 100 (S)	[35]
Water-dispersible vitamin D_3_ in low-fat cheese (milk fat 0.8%)	1690		55 (P) 100 (S)	
Vitamin D_3_ in nanoemulsion in cheese (milk fat 2%)	1	Milk acidification with lactic acid, rennet addition, cutting, centrifugation, storage at 4 °C for 90 d	91 (P) 100 (S)	[43]
Free vitamin D_3_ in cheese (17 MPa, 0.9 P/F ratio)	488	Skimmed milk powder added with anhydrous milk fat and water at specific protein/fat (P/F) ratio, homogenization at 17–150 MPa, calcium chloride addition, coagulation at 85 °C, salting, pressing	52 (P)	[32]
Free vitamin D_3_ in cheese (150 MPa, 2 P/F ratio)	499		49.3 (P)	
Water-dispersible vitamin D_3_ in yogurt (milk fat 3.9%)	nd	Starter inoculation, fermentation, storage at 4 °C for 21 d	97.8 (P) 100 (S)	[34]
Free vitamin D_3_ in yogurt (milk fat 3.9%)			96.6 (P) 100 (S)	
Vitamin D_3_ in W/O emulsion in yogurt	12.5	Addition to fresh yogurt, storage at 4 °C for 20 d	15.97 (S)	[45]
Vitamin D_3_ in W/O emulsion in yogurt + goji berry extract			74.90 (S)	
Water-dispersible vitamin D_3_ in yogurt (milk fat 1.5%)	2.25	Starter inoculation, fermentation, storage in opaque containers at 4 °C for 21 d	100 (S)	[38]
Vitamin D_3_ in oil in yogurt (milk fat 1.5%)			99 (S)	
Water-dispersible vitamin D_3_ in yogurt (milk fat 1.5%)	2.25	Starter inoculation, fermentation, storage in transparent containers at 4 °C for 21 d	86 (S)	
Vitamin D_3_ in oil in yogurt (milk fat 1.5%)			80 (S)	
Vitamin D_3_ in W_1_/O/W_2_ emulsion in yogurt (milk fat 6.15%)	10	Starter inoculation, fermentation, storage at 4 °C for 20 d	50 (S)	[49]
Free vitamin D_3_ in yogurt (milk fat 6.15%)			94 (S)	

(P, S): recovery after processing and storage; (P): recovery after processing; (S): recovery after storage; nd: not reported.

A bread model provided evidence of the effects of acid compounds on vitamin D_2_ degradation. In fact, in rye and wheat bread fortified with vitamin D_2_ from dry yeast, the retention after baking at 170 °C for 60 min was 73% and 85%, respectively. The same retention percentages were observed when free vitamin D_3_ was applied to fortify rye and wheat bread. Rye bread has a lower pH than wheat bread, which was the explanation for the lower retention of vitamin D, due to the acid-catalyzed isomerization to isotachysterol [28]. In another fortification study of bread fortified with free vitamin D_3_, retention was only 40%, but baking was carried out at a higher temperature, i.e., 200 °C for 20 min [68]. For cookies baked at 200–205 °C for a shorter time (12 min), the retention of vitamin D_3_ was 65% [68].

Milk intended for direct use or further processing in dairy products was also considered as a matrix to incorporate either vitamin D_3_ or vitamin D_2_. Nevertheless, contradictory results have been reported in literature regarding vitamin D’s stability in milk upon processing and storage. Natural vitamin D_3_ present in milk was reported to be unstable upon pasteurization and sterilization [57]. Conversely, free vitamin D_3_ added directly to milk before steam injection at 95 °C, spray-drying at 149 °C, and fluid-bed finishing at 107 °C was completely recovered in the dried milk powder [27]. In another study, the percentages of recovery for free vitamin D_2_ in milk after pasteurization at 63 °C for 30 min and sterilization at 121 °C for 15 min were 90 and 67%, respectively [51]. In some applications, both vitamin D_3_ and vitamin D_2_ were previously formulated in water-dispersible forms before addition to milk [37,39]. Pasteurization at 73 °C for 15 s, homogenization at 13.8/3.4 MPa, and storage in opaque plastic containers (with no further specifications) at 4 °C for 21 d did not affect the content of water-dispersible vitamin D_3_ added to milk [37]. Complete retention of vitamin D_3_ was also observed upon UHT processing (138 °C for 2 s) of chocolate milk with 2% added fat [37]. Similarly, water-dispersible vitamin D_2_ was found to be stable in milk upon pasteurization at 63 °C for 30 min and storage at 4 °C for 7 d in glass bottles in the dark, or for 32 h under light (1485–4455 lux), while vitamin D_2_ retention was ~90% when polyethylene pouches were used as a packaging material, regardless of light exposure during storage. This phenomenon was attributed to absorption of vitamin D_2_ by the polymer [39]. Sterilization at 121 °C for 15 min also resulted in total recovery of water-dispersible vitamin D_2_, while the retention during the storage of sterilized milk was not investigated [39].

In another approach, vitamin D_2_ complexed with casein in rCMs was added to milk and found to have improved stability with respect to the free vitamin D_2_ after pasteurization and sterilization, with recovery percentages up to 95 and 76%, respectively [51]. This latter study also reported the effect of storage on vitamin D_2′_s stability in milk packed in glass bottles or low-density polyethylene pouches, which was 70 and 65%, respectively, for vitamin D_2_ in casein complexes, and approximately 45% for free vitamin D_2_ regardless of the packaging material used [51]. Loss of vitamin D_2_ in milk packed in polyethylene pouches was attributed to the porous and hydrophobic nature of this material, which promotes absorption of vitamin D_2_ [39,51]. Moreover, during exposure of milk to light, singlet oxygen is produced, which causes degradation of vitamin D. It is worth noting that, in milk, riboflavin acts as a photosensitizer, thus accelerating the formation of singlet oxygen and leading to the photo-oxidation of vitamin D [75,76].

In cheese, the retention of vitamin D is a key aspect to optimize. In fact, during cheese-making, the fat-soluble vitamin D_3_ could be entrapped in the fat fraction that mostly precipitates in the curd. However, vitamin D_3_ possesses high affinity towards both β-lactoglobulin [77] and α-lactalbumin [78]; hence, it could also be lost in whey [34]. Cheddar cheese obtained from vitamin-D_3_-fortified full-fat milk (3.9% fat, *w*/*w*) showed approximately 90% recovery, regardless of the form in which vitamin D_3_ (free or water-dispersible) was added. The addition of vitamin D_3_ nanoemulsion to partially defatted milk (2.0% fat, *w*/*w*) also resulted in 91% recovery in cheese [43]. Conversely, when water-dispersible vitamin D_3_ was added to reduced-fat milk (0.78%, *w*/*w*), and further processed to Cheddar cheese, the recovery yield was only 55% [35]. Low recovery of vitamin D_3_, equal to 50%, was also observed upon the application of a model cheese-making process, based on heat-induced coagulation at 82 °C in the presence of calcium chloride, regardless of the pressure applied (17–150 MPa) or the protein-to-fat ratio (0.9–2) [32]. During storage, vitamin D_3′_s stability was high for up to 1 year in both full-fat and reduced-fat cheese [35].

Full-fat yogurt was fortified with both free and water-dispersible vitamin D_3_ and stored at 4 °C for 21 d, achieving almost complete recovery with both approaches [34]. Low-fat yogurt was also fortified with both water-dispersible vitamin D_3_ and oil-dispersible vitamin D_3_, and vitamin recovery was found to be complete after 21 d of storage at 4 °C when opaque packaging material was used, while approximately 80% recovery was observed when transparent packaging material was used [38]. However, in another fortification study, a O/W nanoemulsion of vitamin D_3_ made with coconut oil, soybean lecithin, Tween 80, and NaCl was added to yogurt before packaging (packaging material was not specified), and only 15.97% of vitamin D_3_ was recovered after storage at 4 °C for 20 d, unless goji berry antioxidants were added, which allowed 74.90% of vitamin D_3_ to be recovered [45]. Similarly, in a subsequent study, the addition of free vitamin D_3_ to yogurt led to a recovery of 50% after storage (packaging material was not specified), while the addition of a W_1_/O/W_2_ double emulsion made of refined rapeseed oil, polyglycerol polyricinoleate, milk protein concentrate, sodium chloride, and sucrose led to 94% recovery [49]. One possible mechanism for free vitamin D_3_ degradation in yogurt observed in this latter study could be oxidation, triggered by the high fat content (6.5%), whereas the emulsion protected vitamin D_3_ from oxidation.

## 4. Vitamin D in Fortified Foods: Bioaccessibility

Vitamin D is dissolved within the hydrophobic domains of the food matrices—such as bulk oils, fat droplets, or liposomes—or it is associated with proteins [79,80]. The metabolism of vitamin D begins in the stomach, where pepsin plays a role by releasing the fraction of vitamin D associated with proteins, and the gastric lipase hydrolyses vitamin D esters at least partially.

The fundamental step of vitamin D metabolism occurs in the duodenum, where proteases and lipases carry on its release from the food matrix and allow its solubilization in the mixed micelles formed from lipid digestion products, such as free fatty acids, monoglycerides, bile salts, and phospholipids. Then, vitamin D absorption by the epithelial cells takes place, which is mainly protein-mediated at low dietary concentrations and passive at high, pharmacological concentrations of vitamin D [79].

Hence, as for the other fat-soluble vitamins, the bioavailability of vitamin D depends on three phenomena: bioaccessibility (release from the lipid phase of the food matrix and solubilization within mixed micelles); transformation (chemical and biochemical conversion); and transport and uptake (migration through the mucus layer to the surfaces of the intestinal lumen and absorption by epithelial cells) [80]. Among these phenomena, bioaccessibility is the most critical in terms of governing bioavailability [81].

The effect of the food matrix on vitamin D bioavailability is not fully understood. The presence of lipids could improve vitamin D bioavailability by several mechanisms: providing a hydrophobic phase where it can be solubilized; stimulating biliary secretion and, consequently, micelle production; and contributing to micelle formation with their lipid digestion products, inducing chylomicron synthesis, which enhances vitamin D transport outside the enterocytes [79]. Nevertheless, in vivo studies available to date neglect the hypothesis that high amounts of fat in meals improve vitamin D’s bioavailability [44,79].

Dietary fiber seems to impair vitamin D’s bioavailability by affecting micelle formation and by increasing the viscosity of the chime, thus limiting the diffusion of vitamin-D-containing micelles to the brush border [79]. However, in vivo studies do not provide enough data to conclude on the effects of dietary components on vitamin D’s bioavailability.

As discussed previously, data on vitamin D’s bioavailability from food matrices obtained in various studies are difficult to compare because of the different study plans, including subjects involved, amount of vitamin D administered, and duration of the intervention [79]. In particular, vitamin D’s bioavailability is thought to depend on human-associated factors (age, disease, surgery, obesity, genetic variation, etc.) [45]. In vitro studies can provide insights on the effect of the food matrix on vitamin D’s release in the gastrointestinal tract and, hence, support the design of fortified foods (Table 4).

Bread was studied as a food matrix for vitamin D incorporation. Vitamin D_2_ from UV-treated yeast was used for both white and whole bread production, and the resulting bioaccessibility percentages were 15 and 9.9%, respectively. However, the bioaccessibility of free vitamin D_2_ in bread was higher (38%). It was concluded that the yeast cells entrapped vitamin D_2_ and hindered its incorporation into the mixed micelles [30]. As reported above, an in vivo study also led to the conclusion that bread made with UV-irradiated yeast as a source of vitamin D_2_ did not raise serum levels of 25(OH) D, while supplements of both vitamin D_2_ and vitamin D_3_ resulted in a positive effect [18].

Various milk and dairy matrices were used for vitamin D_3_ incorporation. Skimmed milk, partially defatted milk (2% fat), whole milk (3.25% fat), and a standard infant formula powder (4% fat) were fortified with vitamin D_3_ and studied for vitamin D_3_ bioaccessibility [30]. Results showed that, with respect to the initial content, the recovery of vitamin D_3_ in the intestinal digesta was only 40% for skimmed milk, 88% for partially defatted milk, 75% for whole milk, and 77% for infant formula. In general, only a part of vitamin D_3_ found in the digesta was effectively incorporated in the mixed micelles, equal to 70% for whole milk and infant formula and 80% for partially defatted milk and skimmed milk. Hence, considering bioaccessibility as the percentage of vitamin D_3_ recovered in the micellar phase after in vitro digestion compared to the amount of vitamin D_3_ in food, it can be calculated that bioaccessibility was 32, 70, 53, and 54% for skimmed milk, partially defatted milk, whole milk, and infant formula, respectively. It was concluded that bovine milk is an efficient delivery vehicle for vitamin D_3′_s bioaccessibility, even if the optimal amount of lipid for in vivo bioavailability of vitamin D_3_ requires further investigation [30], as observed in vivo [79].

Digestion models have also been tailored to specific populations, because the functioning of the gastrointestinal tract is known to depend on factors such as age and health status [80]. Three in vitro elderly models—namely, oral altered conditions; oral and gastric altered conditions; and oral, gastric, and intestinal altered conditions—were applied in parallel with a healthy adult model to investigate the digestibility of dairy products [82]. Interestingly, vitamin D_3_ naturally present in milk, yogurt, fresh cheese, and aged cheese was equally bioavailable for both the healthy and unhealthy gastrointestinal tract models considered, suggesting that age does not alter vitamin D_3′_s bioaccessibility. On the other hand, vitamin D_3′_s bioaccessibility from milk was found to be 30%, which is lower than that reported previously [30]. Moreover, vitamin D’s bioaccessibility was reported to be greatly dependent on the dairy matrix, being on average 30, 38, 24, and 21% for milk, yogurt, fresh cheese, and aged cheese, respectively [82].

Despite model cheeses not being optimal for vitamin D_3_ supply, gel-like matrices such as cheese can be modulated depending on formulation and processing conditions, leading to different behavior during digestion. In fact, in cheese matrices, aggregated casein micelles create a microstructural network with entrapped solid fat globules and serum, and their digestion is directly related to cheese microstructure [83]. Detailed information on the impact of gel structure on vitamin D_3′_s bioaccessibility was obtained by comparing model acid-coagulated fresh cheeses varying in protein-to-fat ratios (0.9, 1.3, 1.7, and 2) and in the particle size of the cheese milk emulsion due to different homogenization pressures applied (17–150 MPa) [32]. To prepare these model cheeses, milk was fortified by adding free vitamin D_3_ previously dissolved in ethanol and then in melted anhydrous milk fat. Interestingly, vitamin D_3′_s bioaccessibility decreased from 64.51 to 41.56% when the protein-to-fat ratio was increased from 0.9 to 2. An increase in milk homogenization pressure from 17 to 150 MPa also caused a decrease in vitamin D_3′_s bioaccessibility from 64.51 to 27.17%. Both increase in protein content and increase in homogenization pressure caused the formation of a stronger protein network that entrapped smaller fat particles, leading to the hypothesis that changes in the gel microstructure were responsible for the lower release of fat-soluble components during digestion.

**Table 4:** Bioaccessibility of vitamin D from various fortified foods.

Fortified Food	Vitamin D Content (μg/100 g)	Bioaccessibility (%)	Ref
Yeast vitamin D_2_ in white wheat bread	4.67	15	[30]
Yeast vitamin D_2_ in whole wheat bread	4.87	9.9	
Free vitamin D_2_ (control) in whole wheat bread	3.9	38	
Free vitamin D_3_ in skimmed milk	1.12	32	[30]
Free vitamin D_3_ in partially defatted milk (2% fat)	1.02	70	
Free vitamin D_3_ in whole milk (3.25% fat)	1.28	53	
Free vitamin D_3_ in infant powder formula (4% fat)	10.94	54	
Natural vitamin D_3_ in whole milk	3.97	30	[82]
Natural vitamin D_3_ in yogurt	3.1	38	
Natural vitamin D_3_ in fresh cheese	13.8	24	
Natural vitamin D_3_ in aged cheese	21.7	21	
Free vitamin D_3_ in cheese (17 MPa, protein/fat ratio 0.9),	488	64	[32]
Free vitamin D_3_ in cheese (17 MPa, protein/fat ratio 2),	498	51	
Free vitamin D_3_ in cheese (150 MPa, protein/fat ratio 2)	499	27	
Vitamin D_3_ in W_1_/O/W_2_ emulsion in high-protein yogurt	10	100	[49]
Vitamin D_3_ in W_1_/O/W_2_ emulsion in high-protein yogurt	10	100	
Vitamin D_3_ nanoemulsion in oat milk or almond milk	400	20	[46]
Vitamin D_3_ W/O nanoemulsion in almond milk + CaCl_2_ or CaCO_3_	nd	<20	[47]
Vitamin D_3_ in refined olive oil in beef	16.7	5	[33]
Vitamin D_3_ in refined olive oil in semolina or in chickpeas	3.0	25	
Vitamin D_3_ in canola oil in brownies	nd	65.2	[84]
Oily vitamin D_3_ in canola oil + *tert*-butylhydroquinone in brownies	nd	98	
Vitamin D_3_ in zein nanocapsules in a fruit (*Acca sellowiana)* jelly	159	81	[52]

nd: not reported.

The concept of combining vitamin D_3_ fortification of food with increase in protein content is particularly relevant to address the nutritional demands of older people [85]. Consistent with this aim, a high-protein (10%) yogurt fortified with a W_1_/O/W_2_ double emulsion containing berry polyphenols and water-soluble vitamins (C, B_9_ and B_12_) in the inner water phase and lipid-soluble vitamins (A and D) in the oil phase was developed [49]. The ingredients used to formulate the double emulsion were refined rapeseed oil, polyglycerol polyricinoleate, milk protein concentrate, sodium chloride, and sucrose. Free vitamin D_3_ was also added directly to the oil as a control. The results showed that the fortification of yogurt with vitamins loaded into the double emulsion did not hinder the release of vitamins during digestion, which was 100% for both the encapsulated vitamin D_3_ and the free oily vitamin D_3_ at the end of the intestinal phase. However, the kinetics of digestion were different since the release of oily vitamin D_3_ at the end of the gastric phase was considerably higher compared to the release of encapsulated vitamin D_3_.

Plant-based “milk” types were investigated as matrices to incorporate various forms of vitamin D_3_—namely, vitamin D_3_ in O/W nanoemulsion made with corn oil, and vitamin D_3_ nanoemulsion with added TiO_2_ or nanocellulose powders [46]. The vitamin bioaccessibility was around 20% in all of the fortified plant-based milks, indicating that neither type of nanoparticle (inorganic or organic) had a major impact on the bioaccessibility of vitamin D. The reasons for the observed low bioaccessibility were hypothesized to be either a saturation of the mixed micelles or the aggregation and precipitation of the majority of vitamin-loaded mixed micelles due to some of the constituents in the plant-based milks. In a subsequent study, vitamin D_3_ was encapsulated in a nanoemulsion made with corn oil quillaja saponin before addition to almond milk, but this approach also resulted in only 20% vitamin bioaccessibility [47]. Moreover, since plant-based milks are deficient in calcium, either soluble (CaCl_2_) or insoluble (CaCO_3_) calcium salts were added to vitamin-D_3_-fortified almond milk, but the addition of calcium further reduced vitamin D’s bioaccessibility [47]. A negative effect of Ca^++^ on vitamin D_3′_s bioaccessibility had also been observed in a previous study, in which Ca^++^ and vitamin D_3_ had been co-encapsulated in different W_1_/O/W_2_ double emulsions. This effect was attributed to insoluble calcium soap formation in the small intestine step of digestion [86].

In meat, semolina, and chickpeas, vitamin D_3_ was applied after solubilization in refined olive oil, and the resulting bioaccessibility was 5, 25, and 25%, respectively. Low bioaccessibility in meat was attributed to intense oxidation during digestion [33]. Semolina and chickpea antioxidants protected vitamin D_3_ from oxidative degradation; however, despite high vitamin D_3_ content being found in the intestinal digesta of these meals, the incorporation of vitamin D_3_ into the mixed micelles was hindered by tannins, fibers, and saponins [33]. The decrease in vitamin D_3_ bioaccessibility due to the occurrence of oxidation during digestion has also been supported by a previous study, which reported increased bioaccessibility of vitamin D_3_ in brownies fortified with both vitamin D_3_ and *tert*-butylhydroquinone, compared to a control fortified with vitamin D_3_ alone. On the other hand, α-tocopherol and butylated hydroxytoluene were not efficient [84].

A fruit jelly model proved to be an effective matrix for vitamin D_3_ fortification. In fact, vitamin D_3_ encapsulated in zein nanoparticles was used as an ingredient in jelly, which presented 81% bioaccessibility for vitamin D_3_ [52].

## 5. Conclusions and Future Perspectives

For the effective delivery of vitamin D through fortified foods, a careful formulation into nano- and micro-structures to be dispersed in the food matrix is a promising strategy. Due to the complexity and diversity of the food structures, the application of different encapsulation strategies could better address the need to develop carrier systems that simultaneously provide long-term chemical and physical stability, bioaccessibility and, ultimately, in vivo functionality of the vitamin.

However, information on vitamin D’s stability during processing is still fairly correlated with the form in which the vitamin was added to the food matrix. Moreover, knowledge concerning vitamin D’s stability during processing is limited to only a few processing steps, while a better understanding of the kinetic parameters of vitamin D_3_ and D_2_ degradation—i.e., rate constant and activation energy, could allow the prediction of vitamin D content in fortified foods and, thus, enable process optimization under industrial conditions.

Bioaccessibility studies provided insight into the potential efficacy of various vitamin-D-fortified foods, addressing the role of lipids, proteins, fibers, antioxidants, and Ca^++^ ions. The main challenge in future studies will be to acquire advanced knowledge of the interactions between vitamin D and specific molecules—especially single proteins and fibers—in order to take advantages from possible synergism. Moreover, validated in vitro models to assess vitamin D’s bioaccessibility in various target populations—such as the elderly, obese subjects, and subjects affected by malabsorption diseases—could assist a proper fortified food design, since a large variability in response to vitamin D supplementation has also been observed among individuals.
